# Applied anatomy of round window and adjacent structures of tympanum related to cochlear implantation^☆^

**DOI:** 10.1016/j.bjorl.2018.03.009

**Published:** 2018-04-19

**Authors:** Shraddha Jain, Sagar Gaurkar, Prasad T. Deshmukh, Mohnish Khatri, Sanika Kalambe, Pooja Lakhotia, Deepshikha Chandravanshi, Ashish Disawal

**Affiliations:** Jawahar Lal Nehru Medical College, Datta Meghe Institute of Medical Sciences (DU), Department of Otorhinolaryngology and Heand and Neck Surgery, Sawangi(M), Wardha, India

**Keywords:** Round window, Cochlear implantation, Facial recess, Tympanum, Facial nerve, Janela redonda, Implante coclear, Recesso facial, Caixa timpânica, Nervo facial

## Abstract

**Introduction:**

Various aspects of the round window anatomy and anatomy of posterior tympanum have relevant implications for designing cochlear implant electrodes and visualizing the round window through facial recess. Preoperative information about possible anatomical variations of the round window and its relationships to the adjacent neurovascular structures can help reduce complications in cochlear implant surgery.

**Objective:**

The present study was undertaken to assess the common variations in round window anatomy and the relationships to structures of the tympanum that may be relevant for cochlear implant surgery.

**Methods:**

Thirty-five normal wet human cadaveric temporal bones were studied by dissection for anatomy of round window and its relation to facial nerve, carotid canal, jugular fossa and other structures of posterior tympanum. The dissected bones were photographed by a digital camera of 18 megapixels, which were then imported to a computer to determine various parameters using ScopyDoc 8.0.0.22 version software, after proper calibration and at 1× magnification.

**Results:**

When the round window niche is placed posteriorly and inferiorly, the distance between round window and vertical facial nerve decreases, whereas that with horizontal facial nerve increases. In such cases, the distance between oval window and round window also increases. Maximum height of the round window in our study ranged from 0.51–1.27 mm (mean of 0.69 ± 0.25 mm). Maximum width of round window ranged from 0.51 to 2.04 mm (mean of 1.16 ± 0.47 mm). Average minimum distance between round window and carotid canal was 3.71 ± 0.88 mm (range of 2.79–5.34 mm) and that between round window and jugular fossa was 2.47 ± 0.9 mm (range of 1.24–4.3 mm).

**Conclusion:**

The distances from the round window to the oval window and facial nerve are important parameters in identifying a difficult round window niche. Modification of the electrode may be a better option than drilling off the round window margins for insertion of cochlear implant electrodes.

## Resumo

**Introdução:**

Vários aspectos da anatomia da janela redonda e da anatomia da caixa timpânica posterior são relevantes, devido a suas implicações no desenho dos eletrodos para o implante coclear e na visibilidade da janela redonda através do recesso facial. Informações prévias sobre possíveis variações anatômicas da janela redonda e suas relações com as estruturas neurovasculares adjacentes podem ajudar a reduzir as complicações dessa cirurgia.

**Objetivo:**

O presente estudo foi realizado para avaliar as diversas variações da anatomia da janela redonda e sua relação com as estruturas adjacentes, o que pode ser relevante para a cirurgia de implante coclear.

**Método:**

Trinta e cinco ossos temporais normais de cadáveres humanos frescos foram dissecados para avaliação da anatomia da janela redonda e sua relação com o nervo facial, canal carotídeo, fossa jugular e outras estruturas da caixa timpânica posterior. Os ossos dissecados foram fotografados com uma câmera digital de 18 megapixels e as imagens importadas para um computador para determinar diversos parâmetros, utilizando-se o *software* ScopyDoc versão 8.0.0.22, após a calibração adequada e com ampliação de 1×.

**Resultados:**

Quando o nicho da janela redonda se encontra posicionado posteriormente e inferiormente, a distância entre a janela redonda e o nervo facial vertical diminui, enquanto aquela com o nervo facial horizontal aumenta. Em tais casos, a distância entre a janela oval e a janela redonda também aumenta. A altura máxima da janela redonda em nosso estudo variou de 0,51 a 1,27 mm (média de 0,69 ± 0,25 mm). A largura máxima da janela redonda variou de 0,51 a 2,04 mm (média de 1,16 a 0,47 mm). A distância mínima média entre a janela redonda e o canal carotídeo foi de 3,71 ± 0,88 mm (variação de 2,79 a 5,34 mm) e entre a janela redonda e a fossa jugular, em nosso estudo, foi de 2,47 ± 0,9 mm (variação de 1,24 ± 4,3 mm).

**Conclusão:**

A distância da janela redonda a partir da janela oval e do nervo facial são parâmetros importantes para a identificação do difícil nicho da janela redonda. A modificação do desenho do eletrodo pode ser uma opção melhor do que o broqueamento das margens da janela redonda para a inserção dos eletrodos no implante coclear.

## Introduction

There is recent increase in interest in the understanding of the round window anatomy, as it is important for cochlear implant surgery and also in the emerging field of drug delivery to the inner ear.^1^ This was noted as early as 1961 by Sellers, who believed that it is the practicing otologist rather than an experimental scientist, who will have more interest in the re-evaluation of the round window.^2^ The round window membrane (RWM) is located at the end of the scala tympani, where the hook bends postero-medially, and is barely visible on the promontory, being hidden under a bony overhang of the round window niche. In the beginning, the cochlear implant (CI) was inserted through the RWM. This was later almost totally replaced by drilling a cochleostomy to enter the scala tympani.^3^, ^4^, ^5^, ^6^, ^7^ When using the RW approach, the crista fenestra was an obstruction during electrode insertion, and hence, drilling a cochleostomy in front of the RWM was preferred.^6^ This led to positioning of the electrode along the outer wall of the cochlea. Currently more emphasis is being placed on preservation of residual hearing during CI surgery, and the utility of a cochleostomy approach, in this regard is being questioned by several authors.^8^ Many surgeons therefore have returned to the RW insertion technique.^9^ This is now possible through the design of new atraumatic electrode arrays.

The anatomy of the human cochlea is highly variable in terms of dimensions in each individual (“cochlear fingerprint”).^10^ The cochlea begins where the basilar membrane and organ of Corti form the “hook region” near the posterosuperior rim of the round window. The variations in cochlear anatomy begin at the first segment of the scala tympani itself (hook area), in the form of unusual narrowing or constriction. In such a cochlea, for the purpose of cochlear implantation, the drilling of a cochleostomy would be difficult to perform without damaging the lateral wall and spiral ligament. Even a round window insertion could damage the spiral lamina with consequent loss of any residual hearing. The position of the RW niche opening and size and shape of the round window membrane also are variable in each individual and have implications relative to the site and type of electrode insertion.^11^, ^12^, ^13^ Various aspects of the round window (RW) anatomy are relevant for atraumatic insertion of cochlear implant electrodes.^14^

Preoperative awareness of possible anatomical variations of the RW and its relationships to the carotid canal (CC), jugular bulb (JB), facial nerve (FN), and oval window (OW) can help reduce complications in CI surgery.^15^ There have been only a few cadaveric studies done evaluating these parameters in the Indian population. Hence, the present study was undertaken in adult wet cadaveric temporal bones to assess the variations in round window anatomy that may be relevant for cochlear implant surgery and for cochlear implant electrode design. The study also attempted to analyze the topographical relationships of the round window with various middle ear structures with their implications for RW visibility through the facial recess in order to provide information that may reduce neurovascular complications in CI surgery.

## Methods

Thirty-four normal wet human cadaveric temporal bones were studied by dissection for anatomy of round window and posterior tympanum and other anatomical parameters relevant to the cochlear implantation by the facial recess approach. The study was approved by the Institutional Ethics Committee, vide letter no. IEC/2014-15/866 dated 25-09-2014.

Each temporal bone was mounted on the temporal bone holder and using standard otosurgical instruments, dissections were done under magnification from a Carl Zeiss OPMI PICO microscope. The bones were dissected with a motor drill, starting from McEwan's triangle, after the periosteum over the mastoid bone was removed, and the mastoid cortex exposed. Then a complete mastoidectomy was performed. The overlying skin and bony structures of the external auditory canal were removed. Posterior tympanotomy was then performed. For this, first the mastoid segment of the facial nerve was skeletonized, leaving a small incus bridge intact. Then, the chorda tympani was dissected through the takeoff point (mastoid segment) of the facial nerve up to the point when it entered the middle ear. The facial recess was thus delineated. Posterior tympanotomy was then performed with a 0.7 mm diamond burr to visualize the round window niche, round window, stapes and pyramidal eminence after removing the tympanic membrane. The visibility of the round window was assessed through the posterior tympanotomy, after removal of malleus, incus and stapes supra-structure. The RW and OW areas on the medial wall of tympanic cavity were exposed. The shape of the RW was observed. The dissected bones were photographed by a digital camera of 18 megapixels. The photographs were then imported to a computer to determine the following parameters using ScopyDoc 8.0.0.22 version software, after proper calibration and at 1× magnification, in order to find some correlation of those with unfavorable round window anatomy, that might have implications for electrode implantation and the prevention of neurovascular complications. The carotid canal (CC), facial canal (FC), and jugular fossa (JF) were then unroofed and measurements were made of the following parameters:

1. Maximum height of RW (RWh).
2. Maximum width of RW (RWw).
3. Minimum distance between RW and carotid canal (RW-CC).
4. Minimum distance between RW and roof of jugular fossa (RW-JF).
5. Minimum distance between RW and horizontal facial canal (RW-HFC).
6. Minimum distance between RW and vertical facial canal (RW-VFC).
7. Minimum distance between RW and oval window (RW-OW).
8. The distance from pyramidal eminence to horizontal segment of facial nerve (PE-HFN).
9. The distance from pyramidal eminence to vertical segment of facial nerve (PE-VFN).
10. Distance between pyramidal eminence and anterior round window (PE-RW).

### Statistical analysis

The data was analyzed using the MS Office 2007 Excel spreadsheet (Microsoft Corp., Remond, WA) and the program SPSS 22.0 (SPSS Inc., Chicago, IL). Mean, standard deviation (SD), and range of each parameter was computed. By using Pearson's correlation coefficient, positive or negative correlation was analyzed for the various distances between anatomical landmarks in the posterior tympanum and for identification of any unfavorable round window anatomy visible through facial recess.

## Observations and results

### The shape of the RWM

**Figure 1:**
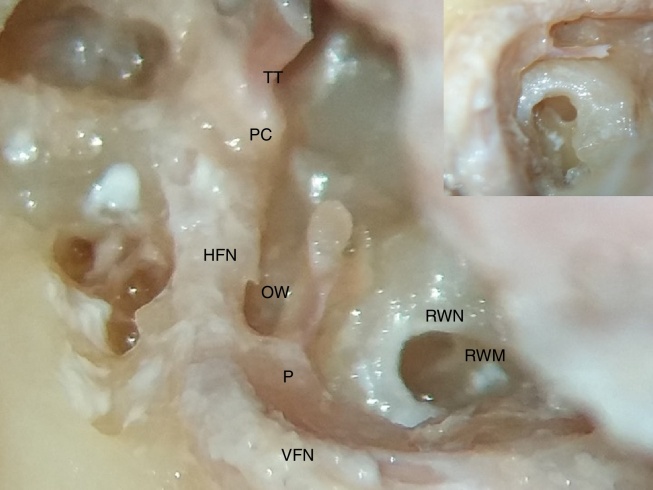
Photograph showing saddle shape of round window membrane (RWM), situated deep in round window niche (RWN). VFN, vertical facial nerve; HFN, horizontal facial nerve; P, pyramid; OW, oval window; PC, processus cochleariformis; TT, tendon of tensor tympani. Inset on right upper corner shows magnified image of round window membrane and stapedius tendon arising from pyramid.

**Figure 2:**
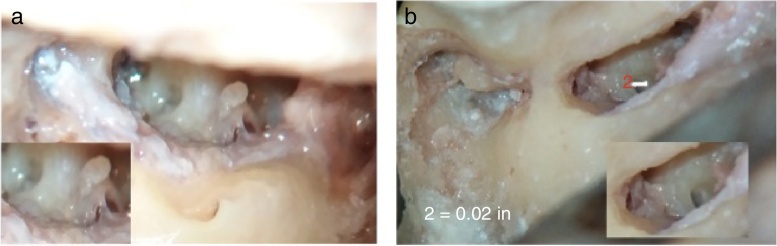
(A, B) Photographs showing saddle shaped round window membrane in A and ovoid in B. In B, round window is seen to be placed more inferiorly and posteriorly, an unfavorable position, but still it can be seen by table adjustment.

In our study, we encountered a saddle-shaped round window membrane in 24 bones; it was ovoid in shape in 8 bones, and triangular in 2 bones (Figure 1, Figure 2; showing different shapes of RWM).

### Maximum height and width of RW (RWh and RWw)

The greatest height of the RW in our study ranged from 0.51 to 1.27 mm with mean of 0.69 ± 0.25 mm. The greatest width of round window ranged from 0.51 to 2.04 mm with mean of 1.16 ± 0.47 mm.

**Figure 3:**
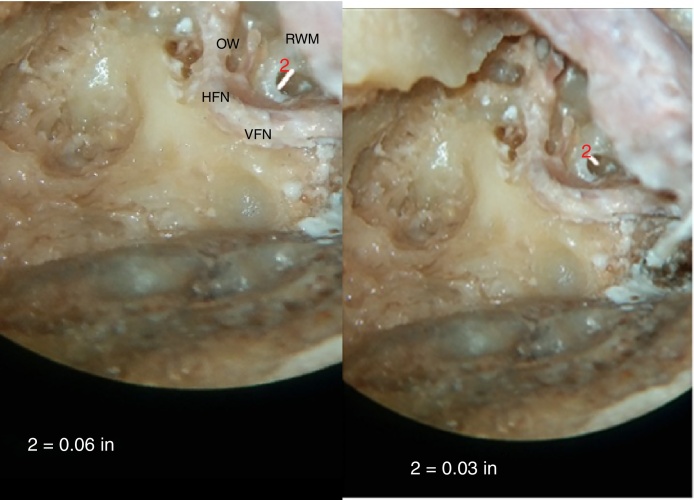
(A, B) Photographs showing measurement of maximum height and width of round window membrane. (A) shows measurement of width, which is in horizontal dimension and (B) shows measurement of height of RWM, which is in vertical dimension. It can be seen that width is more than height of RWM as it is aligned horizontally. RWM, round window membrane; OW, oval window; VFN, vertical facial nerve; HFN, horizontal facial nerve.

**Figure 4:**
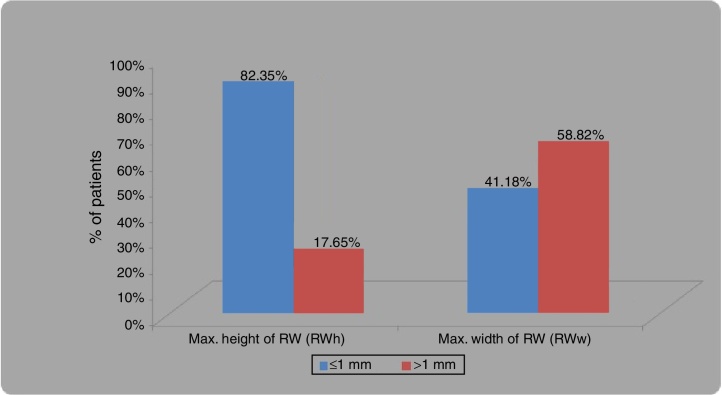
Bar diagram showing frequency of distribution for maximum height of RWM (RWh) and maximum width of RW (RWw), of ≤1 and >1 mm.

A round window height of ≤1 mm was found in 82.35% bones, whereas maximum width of round window of ≤1 mm was found in 41.18% bones, as shown in Table 1 and Figure 3, Figure 4.

**Table 1:** Descriptive statistics for maximum height of RW (RWh) and maximum width of RW (RWw).

	≤1 mm	>1 mm
Max. height of RW (RWh)	28 (82.35%)	6 (17.65%)
Max. width of RW (RWw)	14 (41.18%)	20 (58.82%)

### Distance between RW and carotid canal (RW-CC)

**Figure 5:**
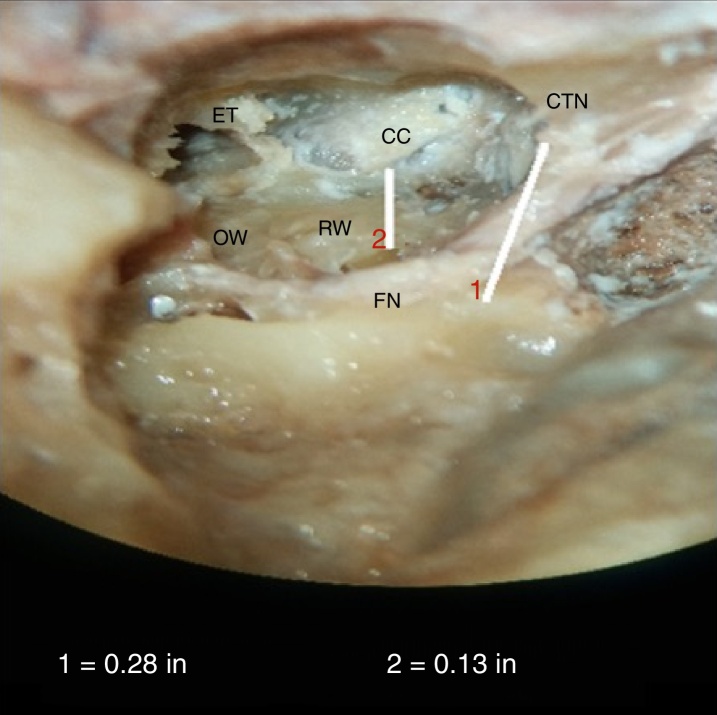
Photograph showing measurement of the minimum distance between round window, RW and carotid canal, CC. FN, facial nerve; OW, oval window; ET, eustachian tube opening; CTN, chorda tympani nerve. Line 1 is for calibration.

Average shortest distance between RW and CC was 3.71 ± 0.88 mm in our study with a range of 2.79–5.34 mm, and the majority of cadavers had distances in the range of 2–4 mm (55.88%); 44.12% had distances in the range of 4–6 mm, as shown in Table 2 and Fig. 5. None of the bones in our series exhibited distance of <2 or >6 mm.

**Table 2:** Descriptive statistics for frequency of distribution of range of minimum distance between RW and CC (RW-CC).

	No. of patients	Percentage (%)
<2 mm	0	0
2–4 mm	19	55.88
4–6 mm	15	44.12
>6 mm	0	0
Total	34	100

### Distance between RW and roof of jugular fossa (RW-JF)

**Figure 6:**
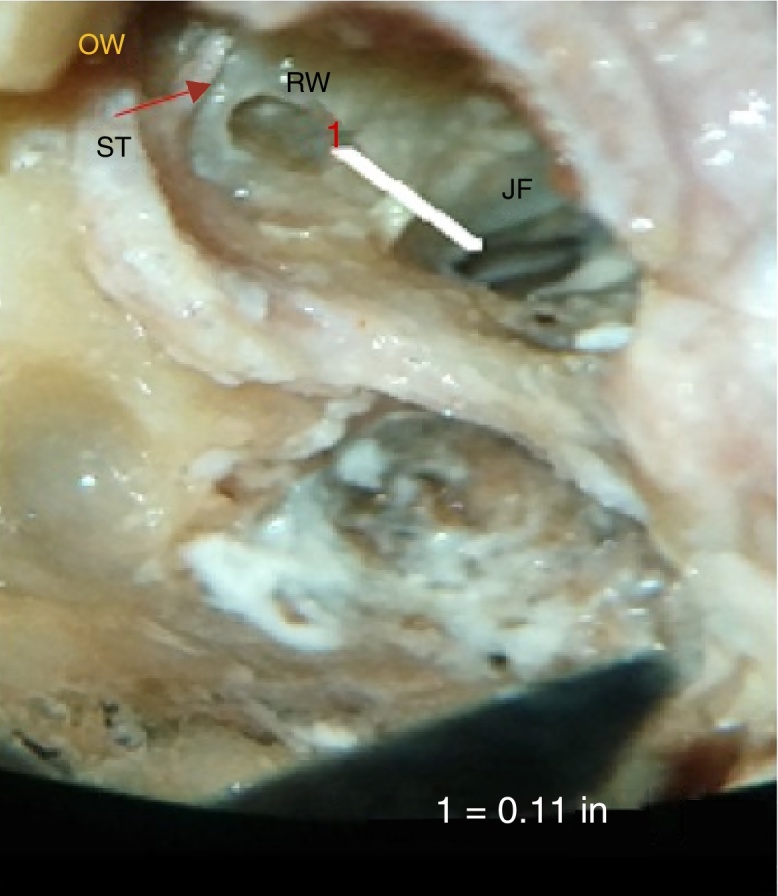
Photograph showing measurement of the minimum distance between round window, RW and jugular fossa, JF. OW, oval window; ST, stapedius sendon.

Average shortest distance between RW and JF in our study was 2.47 ± 0.9 mm with range of 1.24–4.3 mm. The majority of cadavers had distances in range of 2–4 mm (67.65%), and 29.41% had distances of <2 mm, as shown in Table 3 and Fig. 6.

**Table 3:** Descriptive statistics for frequency of distribution of range of minimum distance between RW and JF (RW-JF).

	No. of patients	Percentage
<2 mm	10	29.41
2–4 mm	23	67.65
4–6 mm	1	2.94
>6 mm	0	0
Total	34	100

### Distance from RW to the horizontal facial canal (RW-HFC) and between RW and vertical facial canal (RW-VFC)

**Figure 7:**
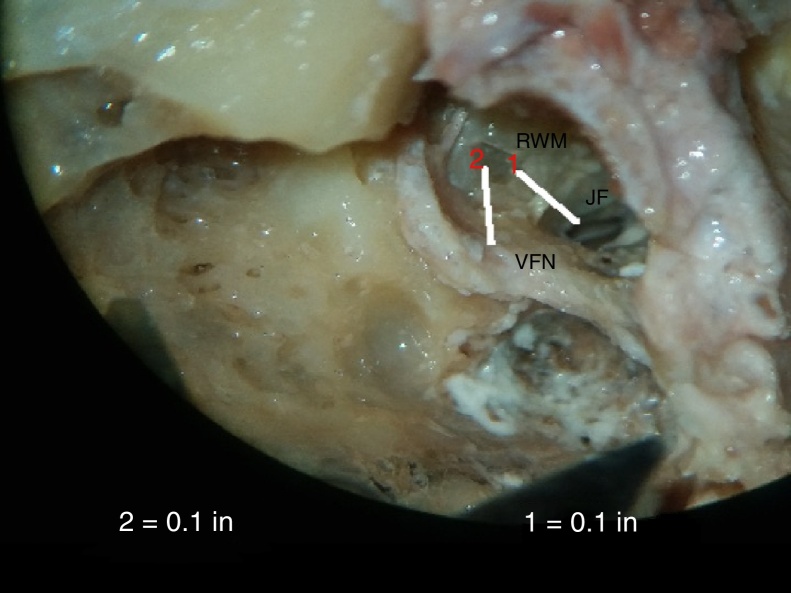
Photograph showing measurement of the minimum distance between round window membrane (RWM), and vertical facial nerve (VFN). Line 1 is for calibration. JF, jugular fossa.

**Figure 8:**
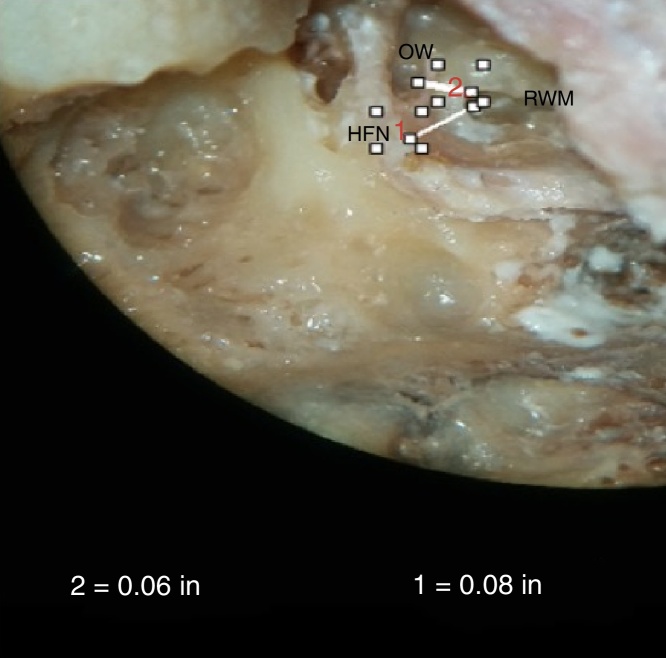
Photograph showing measurement of the minimum distance between round window membrane (RWM) and horizontal facial canal (HFN), and RWM and oval window (OW).

In our study, average shortest distance between RW and the horizontal facial canal was 2.53 ± 0.5 mm (range of 2.03–3.04 mm) and average shortest distance between RW and vertical facial canal (RW-VFC) was 2.11 ± 0.43 mm (range of 1.68–2.54 mm) (Figure 7, Figure 8).

By using Pearson's correlation coefficient, significant negative correlation was found between minimum distance between RW and HFC (RW-HFC) and minimum distance between RW and VFC (RW-VFC) (*r* = −1.00, *p*-value = 0.0001) (Table 4). We found an inverse relationship between the two distances, for unfavorable (posterior and inferiorly placed) RWs, that is, if the distance between round window and vertical facial canal decreased, that with horizontal canal increased.

**Table 4:** Pearson's correlation coefficient between min. distance between RW and HFC (RW-HFC) and min. distance between RW and VFC (RW-VFC).

	Mean	Std. Deviation	*n*	Correlation ‘*r*’	*p*-value
Min. distance between RW and HFC (RW-HFC)	2.53	0.51	34	−1.00	0.0001,S
Min. distance between RW and VFC (RW-VFC)	2.11	0.43	34		

### Minimum distance between RW and oval window (RW-OW)

Mean shortest distance between RW and OW in our study was 2.02 ± 0.56 mm and the majority of cadavers had distances in the range of 1–2 mm (61.76%), with 38.24% having distances in the range of 2–3 mm, as shown in Table 5 and Fig. 8.

**Table 5:** Descriptive statistics for minimum distance between RW and OW (RW-OW).

	No. of patients	Percentage
<1 mm	0	0
1–2 mm	21	61.76
2–3 mm	13	38.24
Total	34	100

### Correlation between the distance from RW to the VFC (RW-VFC) and between RW and OW (RW-OW)

**Figure 9:**
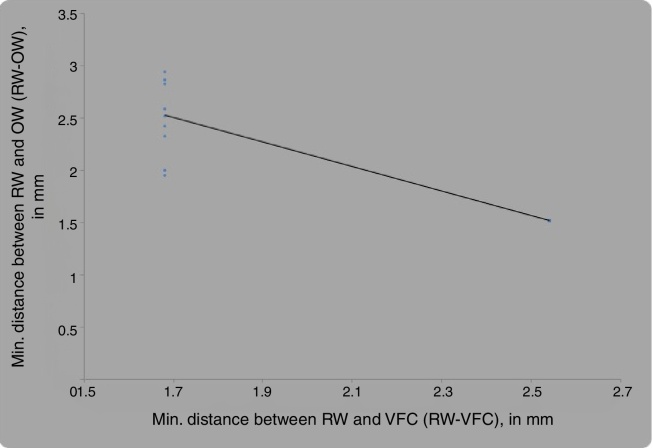
Correlation between minimum distance between RW and VFC (RW-VFC) and minimum distance between RW and OW (RW-OW), by Pearson's correlation coefficient.

By using Pearson's correlation coefficient, significant negative correlation was found between the distance between RW and VFC (RW-VFC) and distance between RW and OW (RW-OW) (*r* = −0.900, *p*-value = 0.0001) (Table 6 and Fig. 9).

**Table 6:** Pearson's correlation coefficient between minimum distance between RW and VFC (RW-VFC) and minimum distance between RW and OW (RW-OW).

	Mean	Std. Deviation	*n*	Correlation ‘*r*’	*p*-value
Min. distance between RW and VFC (RW-VFC)	2.11	0.43	34	−0.900	0.0001,S
Min. distance between RW and OW (RW-OW)	2.02	0.56	34

For unfavorably placed RWM, there exists an inverse relationship between the two distances; that is, if the distance between round window and oval window increases, that of the round window with the vertical facial canal decreases, as it becomes more posterior and inferior.

**Figure 10:**
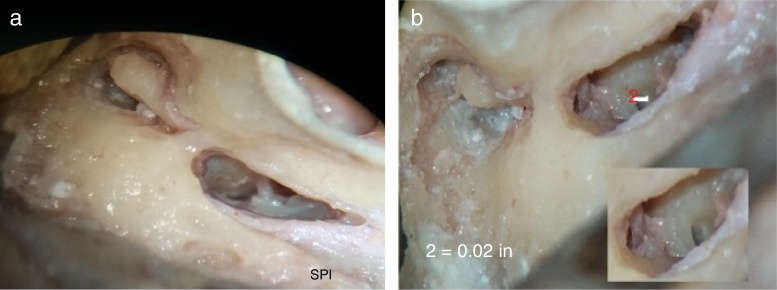
(A) Round window membrane is not visible when making posterior tympanotomy, as can be seen in the photograph. (B) RW visibility through facial recess after table adjustment in posteriorly placed round window membrane.

An unfavorably placed round window membrane could still be visualized through the facial recess by table adjustment (Fig. 10A and B).

### Distance from pyramidal eminence to horizontal and vertical segments of facial nerve (PE-HFN and PE-VFN)

**Figure 11:**
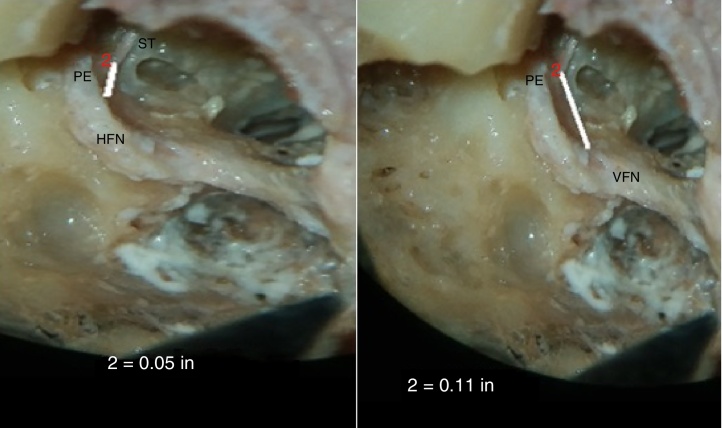
Photograph showing measurement of the minimum distance between pyramidal eminence (PE) to horizontal segment (HFN) and vertical segment (VFN) of facial nerve (PE-HFN and PE-VFN). ST, stapedius tendon.

The average distance from tip of pyramidal eminence to horizontal facial nerve (PE-HFN) in our study was 1.19 ± 0.08 mm and the average distance from tip of pyramidal eminence to vertical facial nerve (PE-VFN) was 2.72 ± 0.21 mm (Fig. 11).

### Distance between pyramidal eminence and anterior round window (PE-RW)

**Figure 12:**
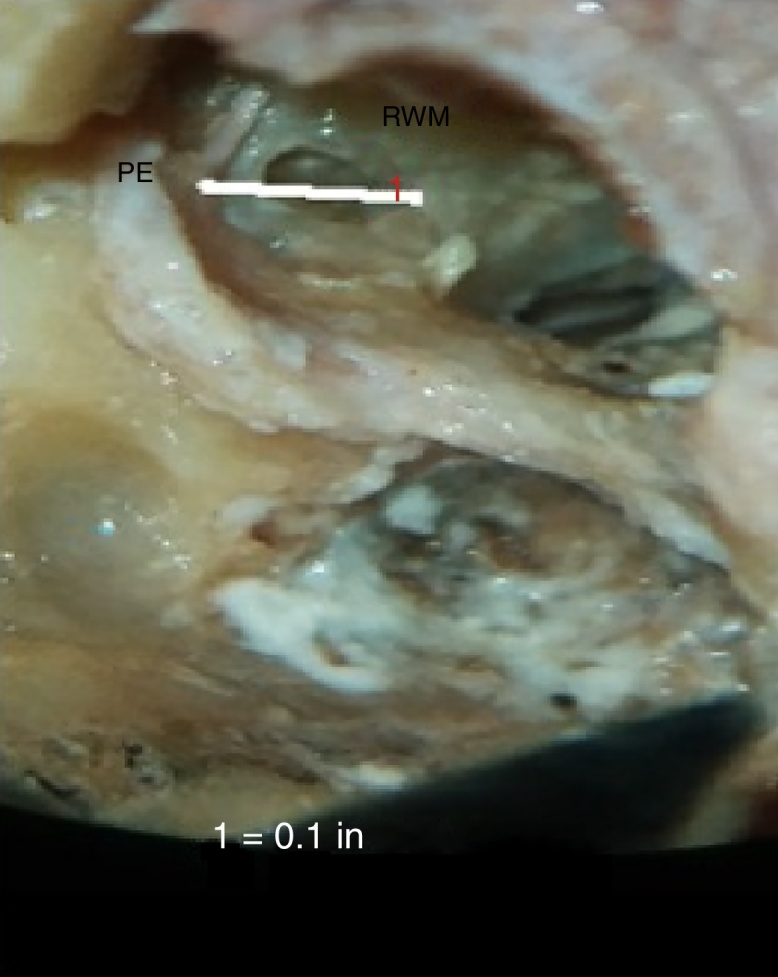
Photograph showing measurement of the minimum distance between pyramidal eminence (PE) and anterior round window (PE-RW).

The average distance between pyramidal eminence and anterior round window (PE-RW) in our study was 2.49 ± 0.1 mm (Fig. 12).

## Discussion

In our study, various parameters of temporal bone anatomy in the middle ear were studied and analyzed for their implications for cochlear implantation by the facial recess approach. The anatomy of the round window niche and round window membrane was studied with respect to shape, size and relationship to various structures in middle ear, firstly for establishing some parameters that could help define difficult cases for round window membrane visibility, and for preventing complications due to injury to adjacent neurovascular structures.

### The shape of the RW

The variations in the anatomy of the human RW may influence the surgical approach and designs of implants aimed at targeting this region.

In our study, mostly we encountered a saddle shape of the round window membrane in 25 bones; it was ovoid in shape in 8 bones, and triangular in 2 bones.

Different authors have found that the human RWM is not round but ovoid with a long and a short diameter. It attains adult dimensions early during fetal development.^16^ RWM has been said to resemble a saddle. Atturo et al. (2014) said that human RW is seldom round but ovoid or orthogonal, skewed, and non-planar (saddle-like).^13^ The membrane is fan-shaped or conical with an antero-inferior and a postero-superior part. Singla et al. (2014) studied human cadaveric temporal bones; the RW shapes in their series were oval (50%), round (20%), triangular (12%), comma (10%), quadrangular (6%), and pear-shaped (2%).^15^

Toth studied the ossification of the round window niche and found that it begins in the 16th week of intra-uterine life and is complete at birth. A process of the otic capsule, called the cartilage bar, forms the inferior wall of the round window niche. The anterior and superior walls of the niche develop by intramembranous ossification, whereas the posterior and inferior walls predominantly form by enchondral ossification.^11^ They concluded that uneven growth of different walls of the round window niche resulted in eight different types of niches: extremely narrow, descending tegmen, anterior septum, bony membrane, open fundus, exostosis, jugular dome and trabeculae.^11^

### Maximum height and width of RW (RWh and RWw)

Maximum height of RW (RWh) in our study ranged from 0.51 to 1.27 mm with mean of 0.69 ± 0.25 mm and the maximum width of round window (RWw) ranged from 0.51 to 2.04 mm with mean of 1.16 ± 0.47 mm. In our study, a maximum height of round window of ≤1 mm was found in 82.35% bones, whereas a maximum width of round window of ≤1 mm was found in 41.18% bones. Mean maximum height of the RW membrane in our study was <1 mm. In our study, vertical dimension was taken as height and horizontal diameter as width. As the round window is placed horizontally, the latter diameter is greater than the former.

Another anatomical study in the Indian population, conducted by Singla et al. (2014), reported the average height and width of RW as 1.62 ± 0.77 and 1.15 ± 0.39 mm, respectively.^15^ The height of the RW, in their study, ranged between 1 and 2 mm in 58% cases, and it was <1 mm in 12% cases. The width ranged between 1 and 2 mm in most specimens (66%) and was <1 mm in 28%. They also observed RWM measurements of <1 mm in many specimens; however, the percentage of such cadavers was less than that in our study. There was a statistically significant correlation (*r* = 0.4, *p* < 0.01) between the height and width in their study. Su et al., also reported diameters of RWM of <1 mm in a significant number of individuals.^16^

Though there is variation in the size of RWM in different individuals, placement of a standard CI array with a maximum diameter of 1.0 mm through the round window membrane is possible in most cases. Several available electrodes have dimensions less than 1 mm and can be used in cases with small size of RWM.^17^ Our results were in concordance with those of Singla et al. (2004), who also pointed out that certain sizes of the round window membrane did not allow use of all the available current electrode sizes.^15^, ^17^ Hence, if we plan round window insertion of an electrode array for Indian population, proper electrode selection is important and designing may need some modification. Otherwise, we could break the electrodes in the attempt to introduce them. In these cases perhaps the use of a slim and soft electrode would be much better than a curved or perimodiolar one that is much harder and does not bend well.

This is the only Indian study, that we could find to compare with our data. Racial variations may exist. Hence, it is important to conduct studies in order to have racial data as well as universal data for comparison.

Atturo et al. (2014) found the mean longest and smallest diameter of RW as 1.90 and 1.54 mm, respectively.^13^ The mean diameter from the crista fenestra was 1.31 mm. The mean area of the RW was 2.08 mm, which varied between 0.99 and 3.20 mm. They stated that the crista fenestrae of the anterior component formed a “doorstep” that could limit the entry to the scala tympani from the RW niche.

Roland et al. observed substantial variability in size of the RW opening available for electrode insertion.^14^ Area measurements of the portion of the RW covered by the vertical segment of the RW membrane ranged from 0.8 to 1.75 mm^2^ in their series. In a microscopic study of the RW region of 30 temporal bones, they found that the bony overhang of the RW niche limited the visibility of the RW membrane during surgery. The RW membrane area visible through a facial recess opening was typically increased by a factor of 1.5–3 times after drilling the overhang and by as much as 13 times when the opening of the RW niche was relatively small. In case irregularities in contour of the RW margin were present, they also proposed drilling of the anterior-inferior margin of the RW. However, they cautioned that, as this region has close proximity to the cochlear aqueduct opening, this should be attempted carefully. They favored RW insertion believing it could be performed in a manner that is potentially less traumatic than the standard cochleostomy insertion. They considered it to be advantageous in cases where hearing preservation is the goal.

Franz et al. also believed that an improved view along the basal turn of the cochlea could be achieved when the antero-inferior overhang and the crista fenestrae was removed.^18^ They believed that only after the removal of the crista fenestrae would there be enough space for the electrode insertion, and the whole width of the scala tympani in the basal turn could be seen.

In our study, even in a narrow RW niche, we could delineate the round window membrane, especially the anterior part of it. It appeared that even without drilling the bony overhangs, adequate exposure of the RWM for electrode insertion could be achieved. The view of the round window membrane, even in a posteriorly-placed niche, could be nicely achieved, by changing the position of the operating table.

Drilling the round window niche, to increase the visibility for electrode insertion, should be avoided. Use of an appropriate electrode design could be a better solution for hearing preservation benefits.

### Distance between RW and carotid canal (RW-CC)

Average minimum distance between RW and CC was 3.71 ± 0.88 mm in our study with a range of 2.79–5.34 mm. In our study, the shortest distance from RW to the carotid canal (RW-CC) in few bones was as close as 2.79 mm. Most cadavers had RW-CC distance in range of 2–4 mm (55.88%), and 44.12% had this distance in the range of 4–6 mm. Our values were lower than those of previous studies.

In the study of Singla et al. (2014), the mean distance between the RW and CC was 8.03 ± 1.55 mm with a wide range (4.39–11.05 mm).^15^ The CC in their study lay very close to the RW in two (4%) cases where the distance was <5 mm. Wysocki and Skarzynski (1998), in their cadaveric study, also reported results (8.08 ± 1.55 mm), which were similar to those of Singla et al. (2014).^19^, ^20^

The RW is an important anatomical landmark for cochlear implant surgery. When performing a cochleostomy antero-inferior to the RW, the knowledge of the precise safe distance of drilling to avoid inadvertent injury to the ICA is desirable, as such injury could be potentially fatal.^21^ The carotid canal (CC) is a potential space that could give rise to tactile sensations similar to those when entering the cochlea and can mislead the surgeon.^22^ Misplacement of an electrode into the CC has been reported by Gastman et al. (2002), Son et al. (2007), Nevoux et al. (2010) and Ying et al. (2013).^22^, ^23^, ^24^, ^25^ The distance between the round window and CC is also important when the implant is inserted into a partially obstructed and obliterated cochlear scala.^20^ The apparent lack of adherence of the carotid artery to its canal laterally may explain why a potential space could be created by an electrode.

### Distance between RW and roof of jugular fossa (RW-JF)

The average shortest distance from the RW to the JF in our study was 2.47 ± 0.9 mm with a range of 1.24–4.3 mm. Most cadavers had a RW-JF distance in the range of 2–4 mm (67.65%), and 29.41% had distance of <2 mm. We did not find any temporal bone presenting distance between RW and JF of <1 mm. In the study by Singla et al., the distance from the RW to the roof of the JF ranged widely from 0.38 to 8.65 mm (mean ± SD, 2.98 ± 1.68 mm).^15^ In their study, in 8% of cases, this distance was <1 mm, so injury to the Jugular Bulb was mentioned as a possible complication during cochleostomy.

### Distance between RW with horizontal facial canal (RW-HFC) and vertical facial canal (RW-VFC)

In our study, the average shortest distance between RW and the horizontal facial canal was 2.53 ± 0.5 mm (range of 2.03–3.04 mm) and average shortest distance from the RW to the vertical facial canal (RW-VFC) was 2.11 ± 0.43 mm (range of 1.68–2.54 mm). We found an inverse relationship between the two distances, that is, as the distance between round window and vertical facial canal decreases, that with horizontal canal increases, and the round window anatomy becomes unfavorable (more posterior and inferior). Our values may be lower than other studies, as we have measured the shortest distance between round window and facial nerve.

Wang et al. (2013), in their cadaveric study found the distance from the round window membrane to the tympanic segment of facial nerve was 3.97 (±0.61) mm.^26^ The distances of round window niche from tympanic and mastoid segment of facial nerve were 4.13 (±0.38) mm and 7.28 (±0.29) mm, respectively, in their study.

The distance between the RW and FC in the study by Singla et al., ranged from 2.99 to 6.3 mm (mean 4.28–0.67 mm).^15^ In 38% of cases, this distance was <4 mm in their study. They did not measure the distance for the two segments of facial canal separately and their values are not of the shortest distance.

Zou et al., in a 2012 study of 20 human temporal bones also studied other parameters of the scala tympani inlet related to cochlear implantation.^27^ They found distance of 6.70 ± 0.19 mm between the facial nerve and the round window niche.

### Minimum distance between RW and oval window (RW-OW)

Mean distance between RW and OW in our study was 2.02 ± 0.56 mm (range of 1.52–2.94), which was comparable to other studies. We observed that the distance between oval window and round window niche was variable, and had an inverse relationship with the round window visibility through facial recess.^28^ Our findings were similar to those of Pendem et al. (2014) who made use of high resolution computer tomography (HRCT) temporal bone measurements on 37 cochlear implant candidates, aged between 1 and 6 years.^28^ They measured the distance between the short process of incus and the round window niche and also, the distance between the oval window and the round window niche on HRCT images. They classified the visibility of round window niche based on the surgical view (i.e. through posterior tympanotomy) during surgery into three types: (1) Type 1, fully visible; (2) Type 2, partially visible, and (3) Type 3, difficult to visualize. They compared the round window visibility through facial recess during surgery with the distance between round window and oval window. The distance between oval window and the round window niche for Types 1, 2 and 3 were 3.2 ± 0.2 mm; 3.8 ± 0.2 mm and 4.4 ± 0.2 mm respectively, and showed statistically significant difference (*p* < 0.01) among them.

Zhu et al. (2008) measured the average distance between oval window and round window niche as 3.74 mm.^29^ Zou et al. (2012) found the distance from stapes to underneath round window as 2.16 (±0.14) mm and distance of 2.11 (±0.18) mm between the stapes and the round window niche.^27^ Wang et al. (2013) found distance from stapes to round window as 3.60 (±0.55) mm.^26^ The values of the distance between oval window and round window in our study was similar to Zou et al. (2012) and lower than other studies.^26^, ^27^, ^29^

### Distance from pyramidal eminence to horizontal and vertical segments of facial nerve (PE-HFN and PE-MFN)

The average shortest distance from tip of pyramidal eminence to horizontal facial nerve (PE-HFN) in our study was 1.19 ± 0.08 mm and the average minimum distance from tip of pyramidal eminence to vertical facial nerve (PE-VFN) was 2.72 ± 0.21 mm. We observed that this relation was fairly constant with little variation. Hence, pyramidal eminence and facial nerve could be used as landmarks for studying the relationship of round window.

Wang et al. (2013) dissected 16 human temporal bones of eight adult cadaveric heads under surgical microscope through facial recess approach and found the distances from pyramidal eminence to tympanic segment of facial nerve and mastoid segment of facial nerve were 1.05 ± 0.09 and 5.63 ± 0.41 mm, respectively.^26^ They observed that the position of certain anatomical structure, like pyramidal eminence, stapes, short crus of incus, and cochleariform process, was relatively constant and that these could be used as reference points in microsurgery of ear.^26^

### Distance between pyramidal eminence and anterior round window (PE-RW)

The average distance between pyramidal eminence and anterior round window (PE-RW) in our study was 2.49 ± 0.1 mm, which was similar to other studies. We did not compare this parameter with round window visibility through facial recess. The location of round window membrane changes with respect to pyramidal eminence, facial nerve and oval window, and the knowledge of this may help identify the difficult cases.

Zhu et al. (2008) found the average distance from pyramidal eminence to the anterior lip of round window niche to be 4.46 mm.^29^ Zou et al. (2012) found the distance between pyramidal eminence and anterior round window was 2.22 (±0.21) mm.^27^ Wang et al. (2013), in their cadaveric study, found the distances from pyramidal eminence to round window was 3.01 (±0.34) mm.^26^

### Relation between different parameters

All the above parameters provide reference values to determine the position of round window for inserting the electrode array into the scale tympani and opening facial recess initially to avoid potential damage to facial nerve and other neurovascular structures during cochlear implant surgery.

In our study, we have measured the shortest distance from the round window to the tympanic and mastoid segments of the facial nerve and also the distance between RW and oval window, for identifying cases with difficult RW visibility through facial recess. We found that if the distance between round window and vertical facial canal decreases, that with horizontal facial canal increases, and the round window anatomy becomes unfavorable, as the round window becomes more posterior and inferior. In such cases, the distance between oval window and round window also increases. We propose that these distances be measured preoperatively on HRCT temporal bone to identify the unfavorable anatomy of round window for visualization though facial recess. Another potential measurement parameter for assessing difficult round window was pyramidal eminence and anterior margin of RWM, which we did not compare with RW visibility through facial recess in our study.

Our findings were in agreement with Pendem et al., with regards to distance between the oval window and round window, with the unfavorable round window showing an increased distance between RW and OW.^28^ Pendem et al. (2014) found the distance between the short process of incus and the round window niche and the distance between the oval window and the round window niche for Types 1, 2 and 3 were 8.5 ± 0.2 and 3.2 ± 0.2, 8.0 ± 0.4 and 3.8 ± 0.2, 7.5 ± 0.2 and 4.4 ± 0.2 mm respectively, and showed statistically significant differences (*p* < 0.01) among them.^28^ Considering short process of incus as a definitive land mark, they measured the distance between the short process of incus and round window niche to identify the variations in position of round window niche. They observed that the visibility of round window niche through posterior tympanotomy became difficult when the distance between the tip of short process of incus (fossa incudis) and the round window niche decreased. This occurs because, when the tip of short process of incus (fossa incudis) is located posteriorly, the round window niche is displaced further posterior and superior (an anatomic variation), and the distance between the short process of incus and the round window niche decreases.

We found that the distance from the round window to the tympanic and mastoid segments of the facial nerve was a better indicator of posterior and inferior displacement of round window and a helpful measurement to avoid intra-operative nerve damage. In such cases, table adjustment is needed to visualize the RWM through the facial recess.

Our findings were similar to those of He et al. (2011), who conducted a study of the anatomy related to cochlear implantation guided by HRCT.^30^ They concluded that the axial distance between facial nerve and posterior wall of external auditory canal and the distance from facial nerve to round window in the semi-longitudinal plane were the most important parameters to identify the position of facial nerve. According to them, preoperative measurements related to the vertical portion of facial nerve, posterior wall of external auditory canal and round window by HRCT could be a helpful guide in CI surgery.

Our results were also in concordance with the results of Park et al. (2015), that a thicker round window bony overhang was not associated with greater difficulty in accessing round window.^31^ It is the orientation and size of the round window (i.e. how posterior and inferior it is), rather than thickness of the bony overhang, that better predicts the difficulty with round window access.

## Conclusion

Prior imaging of the distance from round window to horizontal facial canal, and of round window to oval window can help predict the difficulty of round window exposure as can the size of the facial recess.

Even with a narrow RW niche, the round window membrane can be delineated, even the anterior portion and adequate exposure of the RWM for electrode insertion can be obtained without drilling the bony overhang.

Round window electrode insertion is feasible in most cases by a facial recess approach, but the small-sized round window membranes would not allow all the currently available electrode sizes on the market to be used. Rather than drilling the round window niche, for atraumatic electrode insertion, modification of electrode design to make available slimmer and softer electrodes could be a better solution for hearing preservation.

The view of the round window membrane, even in posteriorly placed niche can be nicely achieved, by changing the position of the table.

The relation between pyramidal eminence and facial nerve is fairly constant without much variation. It is the orientation of RWN and membrane that changes. Hence, pyramidal eminence and facial nerve can be used as landmarks for studying the relationship of round window.

## Areas of future research

The parameters of relation of round window with neurovascular structures are of cadaveric dissection limits, which are more than those in real patients, and hence need to be compared with imaging studies, in Indian population, to confirm the above findings.

Radiologic studies to confirm the above findings.

## Conflicts of interest

The authors declare no conflicts of interest.
